# The role of C4d and donor specific antibodies in face and hand transplantation—a systematic review

**DOI:** 10.3389/frtra.2024.1442006

**Published:** 2024-09-03

**Authors:** Lioba Huelsboemer, Jake Moscarelli, Alna Dony, Sam Boroumand, Alejandro Kochen, Leonard Knoedler, Catherine T. Yu, Sacha C. Hauc, Viola A. Stögner, Richard N. Formica, Christiane G. Lian, Georg F. Murphy, Bohdan Pomahac, Martin Kauke-Navarro

**Affiliations:** ^1^ Division of Reconstructive and Plastic Surgery, Yale School of Medicine, New Haven, CT, United States; ^2^ School of Medicine, University of Leeds, Woodhouse, Leeds, United Kingdom; ^3^ University of Regensburg, Regensburg, Germany; ^4^ Department of Medicine, Section of Nephrology and Transplantation, Yale School of Medicine, New Haven, CT, United States; ^5^ Program in Dermatopathology, Department of Pathology, Brigham and Women’s Hospital, Harvard Medical School, Boston, MA, United States

**Keywords:** C4d, donor specific antibody, vascularized composite allotransplantation, VCA, reconstructive surgery, antibody-mediated rejection

## Abstract

To date, little is known about the mechanisms of rejection in vascularized composite allotransplantation, particularly for antibody mediated rejection. Additionally, no clear guidelines exist for the diagnosis and management of antibody-mediated rejection in vascularized composite allotransplantation. A systematic review of electronic databases (Embase and PubMed) was conducted to evaluate the relationship of donor specific antibodies and C4d deposition in correlation with cellular rejection following hand and face transplantation reported by centers between 1998 and July 2023. We extracted data on serum donor specific antibodies at the time of biopsy proven rejection according to Banff classification and C4d staining of target tissues. Mann-Whitney U tests were performed to compare rejection grade between groups divided by status of C4d deposition and serum donor specific antibodies, and Fisher's Exact test was used to assess association between the two markers. This review adhered to PRISMA guidelines. A total of 26 patients (5 face, 21 hand) were identified and data on 90 acute rejection episodes with information on Banff grade, donor specific antibody status, and C4d deposition were available. Donor specific antibodies were found to be associated with higher rejection grade (*p *= 0.005). C4d was not found to be associated with higher rejection grade (*p *= 0.33). Finally, no significant association was found between concurrent status of the two markers (*p *= 0.23). These findings suggest that the presence of donor specifc antibodies may be associated with higher grades of acute cellular rejection following hand and face transplantation. More consistent reporting on rejection episodes is needed in order to better understand antibody-mediated rejection in vascularized composite allotransplantation.

## Introduction

Despite clear benefits of vascularized composite allotransplantation (VCA) in transforming the lives of patients, this relatively novel development in transplantation is associated with unique and often challenging complications (1, 2). These include repeated acute cellular rejection, chronic rejection, antibody mediated rejection (ABMR), and ischemia-reperfusion injury, which are all major hurdles in solid organ transplantation (SOT) as well (3–6). In the latest Banff classification from 2019, ABMR is outlined for kidney transplants (7). However, similar to chronic rejection, ABMR has not yet been clearly defined for VCAs. Nonetheless, isolated cases have been reported (7–9). The definition of ABMR in renal transplant recipients is subdivided into active ABMR and chronic ABMR. Active ABMR has to meet the following three criteria: 1. Histologic evidence of acute tissue injury (microvascular inflammation/intimal or transmural arteritis/acute thrombotic microangiopathy/acute tubular injury), 2. Evidence of current or recent antibody interaction with vascular endothelium (including C4d staining/microvascular inflammation/increased gene expression associated with ABMR) and 3. Serologic evidence of circulating donor specific antibodies (DSA). Chronic active ABMR is defined by the following criteria: 1. Morphologic evidence of chronic tissue injury (including transplant glomerulopathy/severe peritubular capillary basement membrane multilayering/arterial intimal fibrosis of new onset), plus criteria 2 and 3 for active ABMR above (3). During ABMR, DSA activate the classical pathway of the complement system, resulting in the cleavage of C4d. This leads to endothelial deposition of C4d which can serve as an informative marker for complement activation (8, 9). Histologic evidence of acute tissue injury, linear C4d staining in peritubular capillaries or medulla vasa recta, and circulating donor specific antibodies to HLA or other antigens, are hallmarks of ABMR in SOT, and the presence of both C4d and DSA have been shown to be associated with a higher risk of allograft loss in renal transplantation (10). Since the current Banff Working Classification 2007 of Skin-Containing Composite Tissue Allograft Pathology does not contain explicit criteria for the diagnosis of chronic rejection and ABMR (11), the diagnostic utility of DSA and C4d in VCA still remains uncertain. Various studies have demonstrated a high prevalence of DSA in VCA rejection, and the presence of DSA was found to correlate significantly with the number of rejection episodes observed per year in hand transplant patients (12–15). As such, monitoring DSA may be an effective way to assess rejection risk. C4d deposition has not yet proven to be an effective marker of ABMR in VCA, with staining often appearing to be nonspecific (16). The goal of this systematic review is to explore the association of C4d and DSA with acute rejection of VCA based on the currently available data. We specifically aim to investigate any association between C4d and DSA, as well as any relationship between marker status and acute rejection severity. This review focuses on hand and face transplants from established VCA centers worldwide.

## Methods

### Search and screening strategy

**Figure 1:**
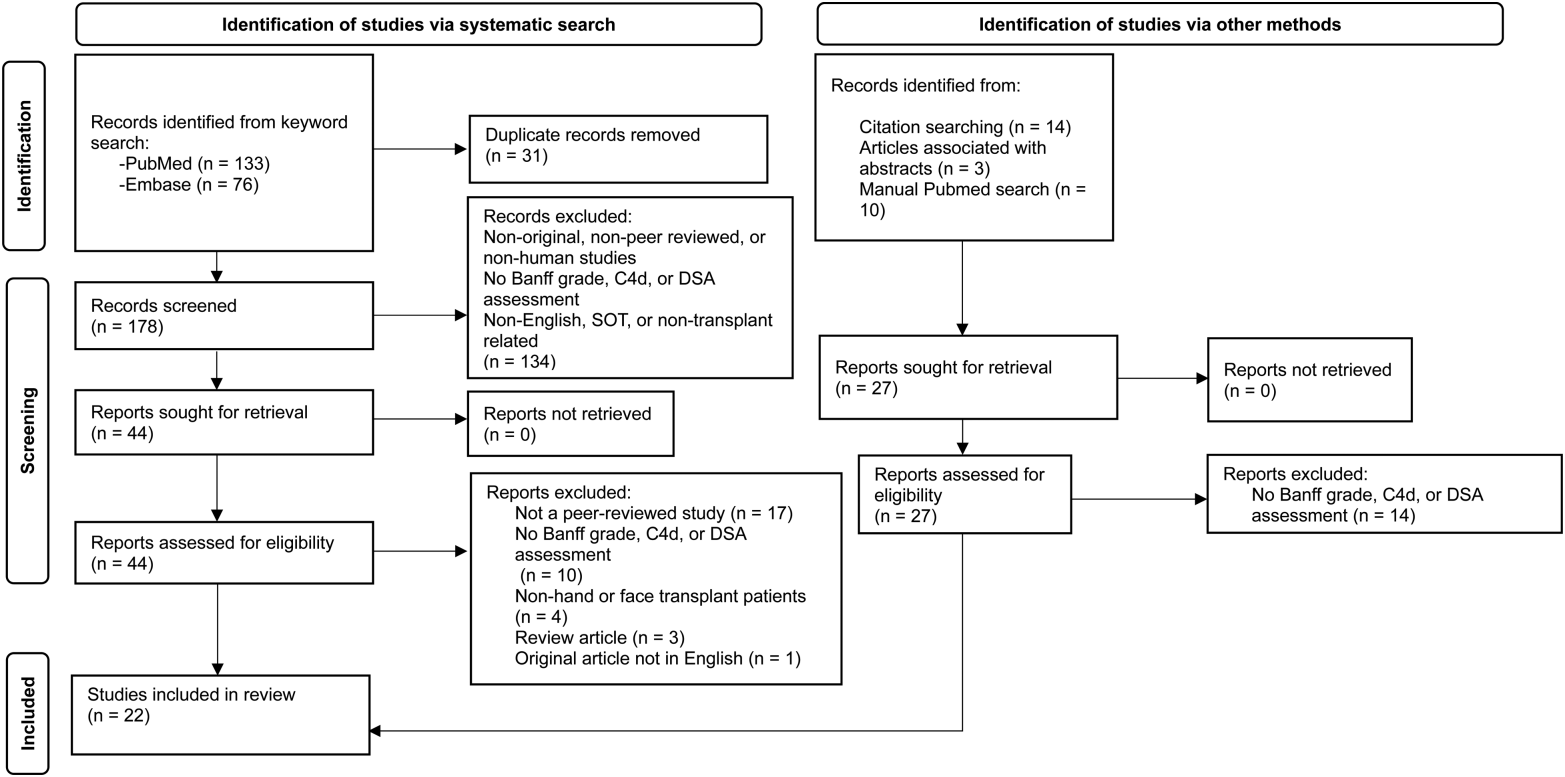
Preferred reporting items for systematic reviews and meta-analyses (PRISMA) flow diagram depicting systematic review and screening results.

This systematic review was performed in accordance with the Preferred Reporting Items for Systematic Reviews and Meta-Analyses (PRISMA) guidelines and is illustrated in Figure 1. PubMed and Embase were searched on July 31st, 2023, using the following keywords: [(C4d OR DSA OR donor specific antibodies) AND (VCA OR vascularized composite allotransplant* OR vascularized composite allograft* OR composite tissue allograft*)]. Articles referenced in papers that met criteria for inclusion, which were not yielded in the original search and may have reported potentially relevant data for extraction, were also considered for inclusion. PubMed was also manually searched for articles that met inclusion criteria but were missed by the original search. Only original, peer-reviewed, human studies performed at active VCA centers were included. Articles had to report acute rejection episodes for hand or face transplants with Banff grade and assessment of C4d or DSA in order to meet criteria for inclusion. Non-English and SOT or non-transplant related studies were excluded. Articles were independently evaluated by two reviewers (J.M.; A.D.) at each stage of screening and any disagreements were resolved by a third independent reviewer (L.H.). Data extracted from each article included transplant type, number of patients in the study with extractable data, country of the VCA center, Banff grade for acute rejection episodes, and C4d and DSA testing and status for each episode. Data extraction was performed by one reviewer (J.M.) and checked-over by a second reviewer (A.D.), and any disagreements were resolved by a third independent reviewer (L.H.).

### Statistical analysis

All statistical analyses were performed in GraphPad Prism for macOS (Version 10.0.2). Only episodes where C4d or DSA status was reported concurrently with Banff grade were utilized in the analysis. In the case that rejection was reported between two grades (i.e., grade I-II), the higher grade was chosen for purposes of analysis. Differences in rejection grade between groups divided by C4d and DSA status alone were assessed with the Mann-Whitney U test, and association between C4d and DSA was assessed with the Fisher's Exact test. Significance level was set at α = 0.05 for all analyses.

## Results

There were 22 articles that met criteria for inclusion in this study and all were assessed for quality using a modified Joanna Briggs Institute (JBI) Checklist for Case Reports (Tables 1, 2) (17–38). Data was extracted from 26 patients, 5 of whom underwent face transplantation, and 21 of whom underwent hand transplantation. A total of 90 episodes of acute rejection were reported along with grading according to the Banff Classification 2007 and details of the assessment and status of C4d, *de-novo*/pre-existing DSA, or both. *De-novo* DSA (defined as serologic evidence of circuluating DSA post-transplantation) was reported in 81 episodes. Testing for pre-existing DSA (defined as serologic evidence of circuluating DSA pior to transplantation) was reported in 12 patients. In six patients pre transplant DSA were identified while in six patients pre transplant DSA were negative. C4d staining of skin biopsies was assessed in 34 episodes, and both C4d and DSA were assessed in 26 episodes. Immunohistochemical assessment of C4d in tissue biopsies was reported at varying levels of detail, and specific anatomical location of positive staining within tissue samples was not considered in this study. Reported rejections ranged from grade 0—IV with a median rejection grade of II. Finally, there were 6 instances of graft failure in the assessed studies. Three of those cases were positive for both C4d and DSA. One case showed nonspecific C4d staining and was negative for DSA. One was C4d positive and did not report on DSA. The last case did not report on C4d and was DSA positive.

**Table 1:** Transplant and rejection episode details.

Source	No. of patients	VCA	Location of VCA center	No. of reported rejections with Banff grade (if available)	No. of rejections w/assessment of C4d	No. of C4d (+) samples	No. of rejections w/ assessment of DSA	DSA post-transplant	Episodes of De-Novo DSA (+)	Additional assessment	Pre-transplant DSA	Rejection treatment	Diagnosed ABMR	Graft loss	Follow-up time	Graft failure
(17)	1	Hand—bilateral	Pennsylvania, USA	I: 1	0	Not assessed	2	DR15, DR51	2	Negative B and T cell crossmatch	Negative	Topical Prograf	No	No	1 year	No
				II: 1						Cytokines and chemokines: transient increase of chemokines CXCL9 (MIG), CCL4 (MIP-1β) and the cytokine IL-7 at day 8 post-transplant						
(18)	1	Hand	Atlanta/Durham, USA	0: 1 I: 2 I-II: 1 II: 1	3	Negative	2	B48, 60, 61, 81	1	PRA score of 0	N/A	Prednisone Bolus oral; Methylprednisone Bolus IV; Rabbit-anti-thymocyte globulin IV; IVIG and Plasmapharesis; Belatacept intravenous (IV) monthly and sirolimus aiming at trough levels of 8–12 ng/ml. MMF and tacrolimus were discontinued. Prednisone was continued.	Yes	No	42 months	No
(19, 22)	1	Face	Boston, USA	I: 2	18	10	16	A2, A32, B18, B57, DR7, DQ7 and DQ9	10	PRA score of 98	A2, A32, B57, DQ7, DQ9 and DR7	Eculizumab once a week; Steroid Bolus; IVIG; TPE; Bortezomib; extra corporal Photopheresis; ATG; Alemtuzumab	Yes	Yes; Chronic rejection	6 months	Yes; C4d (+) and DSA (+) at the time of graft failure
				II: 4						Positive B and T cell crossmatch						
				III: 12												
				IV: 1												
(22)	1	Face (Re-transplant)	Boston, USA	III: 2	N/A	Negative	0	Negative	0	PRA score of 0	Negative	Predsnisone Bolus; Alemtuzumab	No	No	6 months post retransplantation	No
(20)	1	Face	New York, USA	0: 1 0-I: 1	2	2	1	N/A	0	PRA score of 0; CDCXM: negative for T and B cells. FCXM: negative for T cells but repeatedly positive for donor B cells.	Negative	N/A	No	No	2 years	No
(21, 37, 38)	5	Hand	Innsbruck, Austria	Patient 1–4 episodes	N/A	Positive- Not specified if concomitant with banff graded rejection	N/A	No	0	PRA score of 5%	N/A	Thymoglobulin; alemtuzumab; rituximab; Immunoadsorption; plasmapheresis	No		20 years	No
				Patient 2–12 episodes				Yes	4	PRA score of 0			Yes		17 years	No
				Patient 3–9 episodes				Yes	0	PRA score of 0			No		14 years	No
				Patient 4–1 episode				Yes	8	PRA score of 0			Yes	Yes; Chronic rejection	7 years	Yes; C4d (+) and DSA (+) at the time of graft failure
				Patient 5–3 episodes				No	0	PRA score of 0			No		5 years	No
(26)	1	Face	Lyon, France	II: 2 III: 2	1	1	2	DBR5, DQ6	5	Flow magnetic resonance imaging (MRI) with a sequence 3D Phase Contrast (3D PCA) of the facial graft showed a decrease in flow of the right facial artery at distal level	N/A	Steriod Bolus; IVIG; Plasmapharesis; Bortezomib; Eculizumab	Yes	Yes; Chronic rejection	10 years	Yes; C4d (+) and DSA (+) at the time of graft failure
(23)	2	Hand—bilateral	Valencia, Spain	I: 2	4	7	4	N/A	0	Biopsy specimen immunostaining also included CD3 (Dako, Denmark) to demonstrate T-cell infiltration and CD19 (Dako) to demonstrate B-cell infiltration	Negative	Prednisone bolus	No	No	7 and 18 months	No
				II: 2												
(24)	1	Face	Montreal, Canada	I: 3	2	Negative	0	N/A	0	Immunophenotypic characterization of the lymphoytic infiltrate and number of CD3, CD4, CD8, CD20, and CD68+	N/A	Prednisone Bolus; Topical Tacrolimus; Basiliximab	No	No	430 days	No
(25)	6	Hand -bilateral (4), unilateral (2)	Leeds, UK	I: 1	0	Not assessed	25	Negative	0	cPRA score 0 for 5 patients, patient 6 scores 74, lymphocyte subset analysis, B and T cell crossmatch	Negative	Increased immunosuppression, not further specified	No	No	10 months—7 years	No
				II: 16												
				III: 8												
(33)	1	Arm—bilateral	Mexico City, Mexico	II:2 III: 1	0	Not assessed	1	DQ2	1	PRA score of 7%, Negative B and T cell crossmatch	Negative	Tacrolimus, topical steroids, bolus of methylprednisone	No	No	18 months	No
(34)	1	Forearm—bilateral	Mexico City, Mexico	I: 1 II: 3	0	Not assessed	1	CW10, A33, DR52	1	PRA score of 33%; negative B and T cell crossmatch	N/A	Topical IS (not specified), Prednisone bolus	No	No	2 years	No
(35)	1	Transhumeral arm—bilateral	Valencia, Spain	III: 3	3	0	3	Negative	0	PRA score <20%	N/A	Prednisone bolus, Alemtuzumab during 2nd AR that was steroid resistant, topical Tacrolimus	No	No	2 years	No
(36)	6	Hand -unilateral distal forearm (4), hand (1), bilateral hand (1)	Louisville, USA	Patient 1–3 episodes	Not specified	Negative	3	Negative	0	Deep tissue biopsies showed evidence of some intimal hyperplasia in 4 patients	N/A	Prednisone Bolus			12 years	No
				Patient 2–5 episodes			5	Positive	1			Antithymocyte Globulin	Yes		10 years	No
				Patient 3–3 episodes			3	Negative	0			Topical Tacrolimus & steroids			4 years	No
				Patient 4–4 episodes			4	Positive	1			Topical Tacrolimus & steroids	Yes	Yes; Ischemia	9 months	Yes; C4d nonspecific and DSA (-) at the time of graft failure
				Patient 5 – II: 4			4	Negative	0			N/A			2 years	No
				Patient 6 – II: 2			2	Negative	0			IVIG, plasmapheresis and a switch from MMF to Rapamycin			6 months	No
(30)	1	Hand—unilateral	Lyon, France	4 episodes	N/A	1	4	Negative	0	Immunohistochemistry, deep tissue biopsies	N/A	Topical Tacrolimus & steroids	No	Yes; vasculopathy as a result of non-adherence	13 years	Yes; C4d (+) and DSA not reported at the time of graft failure
(31)	5	Hand—bilateral	Lyon, France	Patient 1 – II: 2	N/A	Not assessed	2	Negative	0	Negative B- and T cell crossmatch in all patients; yearly and pre transplant blood lymphocyte subsets	N/A	Oral steroids plus topical tacrolimus	No	No	13 years	No
				Patient 2 – II: 3			3	Positive	1			Oral steroids plus topical tacrolimus			10 years	
				Patient 3 – II: 3 III: 3			8	Negative	0			Prednisone Bolus, ATG, Campath-1H, Photochemotherapy plus topical tacrolimus			6 years	
				Patient 4 – II: 1			1	Negative	0			Oral steroids plus topical tacrolimus			5 years	
				Patient 5 – II: 3			4	Positive	1			Oral steroids, prednisone bolus plus topical tacrolimus			3 years	
(27)	1	Face & bilateral hand	New York, USA	Not detected	N/A	Not assessed	0	B27, DP4, DP23	1	Negative Donor-recipient complement-dependent cytotoxicity, negative T- and B-cell crossmatch	Positive	Plasmapheresis, IVIG during increase of DAS POD 7 and 8	No	No	8 months	No
(32)	1	Hand	Melbourne, Australia	II: 1	0	Not assessed	0	Negative	0	PRA score of 6%, negative B and T cell crossmatch	Positive	Topical Tacrolimus and Clobetasol	No	No	2 years	No
(28)	7	Face	Paris, France	Patient 1 – I: 1 II: 4 III: 2	0	Negative	0	Positive	2	Negative B- and T cell crossmatch in all patients, all but patient 1 presented with PRAs	2 patients positive	IGIV	No	No	9.2 years	No
				Patient 2 – I: 1 III: 1	N/A	Not assessed	0	Negative	0			Prednisone Bolus	No	No	7.1 years	
				Patient 3—none detected	0	Negative	2	Positive	2			IGIV	No	No	65 POD, died due to pseudomonas infection altered transplants	
				Patient 4: II: 1, III: 2 IV:1	N/A	Not assessed	Multiple, not specified, at least 1	Positive	2			Prednisone Bolus, Plasma exchange plus tiruximab, IGIV, ATG	No	No	6.7 years	
				Patient 5—grade II: 2, grade III: 1	N/A	Not assessed	2	B51	2			Prednisone Bolus, ATG	Yes	Yes due to chronic rejection	8 years	
				Patient 6—grade II: 1	N/A	Not assessed	0	Negative	0			Prednisone Bolus	No	No	5 years	
				Patient 7—none detected	N/A	Not assessed	0	Negative	0			N/A	No	No	3.5 years (Suicide)	
(29)	1	Face—retransplant	Paris, France	I: 1 II-III: 2	0	Negative	1	Positive	1		Positive	Prednisone Bolus, Eculizumab	Yes	No	2 years	Yes; C4d not reported and DSA (+) at the time of graft failure

ABM, antibody mediated rejection; ATG, anti-thymocyte globulin; DSA, donor specific antibodies; IS, immunosuppressants; IV, intravenously; IVIG, IV immunoglobuline; MMF, mycophenolate mofetil; N/A, not available; No., number; POD, post operative day; PRA, panel reactive antibodies; TPE, therpaeutic plasma exchange; VCA, vascularized composite allotransplantation.

**Table 2:** Appraisal of study quality using a modified Joanna Briggs Institute (JBI) checklist for case reports.

Source	Demographic characteristics described	Patient's history described	Current clinical condition described	Pre-intervention testing/results clearly described	Intervention(s)/treatment procedures described	Post-intervention testing/results clearly described	Post-intervention clinical condition described	Adverse events/unanticipated events described	Takeaway lessons described
19	Yes	Yes	Yes	Yes	Yes	Somewhat	Yes	Yes	Yes
20	Yes	Yes	Yes	Yes	Yes	Somewhat	Yes	Yes	Yes
9, 21	Yes	Yes	Yes	Yes	Yes	Yes	Yes	Yes	Yes
22	Yes	Yes	Yes	Yes	Yes	Somewhat	Yes	Yes	Yes
23, 37, 38	Yes	Yes	Yes	Yes	Yes	Yes	Yes	Yes	Yes
8	Yes	Yes	Yes	Yes	Described elsewhere	Somewhat	Yes	Yes	Yes
24	Yes	No	Yes	Yes	Yes	Yes	Yes	Yes	Yes
25	Yes	Yes	Yes	Yes	Yes	Yes	No	Yes	Yes
26	No	No	Yes	Yes	Yes	Yes	Yes	Yes	No
33	Yes	Yes	Yes	Yes	Yes	Somewhat	Yes	Yes	Yes
34	Yes	No	Yes	Yes	Yes	Somewhat	Yes	Yes	Yes
35	Yes	Yes	Yes	Yes	Yes	Somewhat	Yes	Yes	Yes
36	Yes	Yes	Yes	Yes	Yes	Somewhat	Yes	Yes	Yes
30	Yes	No	Yes	Somewhat	Somewhat	Somewhat	Yes	Yes	Yes
31	Yes	Yes	Yes	Yes	Yes	Yes	Yes	Yes	Yes
27	Yes	Yes	Yes	Yes	Yes	Yes	Yes	Yes	Yes
32	Yes	Yes	Yes	Yes	Yes	Somewhat	Yes	Yes	Yes
28	Yes	Yes	Yes	Yes	Yes	Yes	Yes	Yes	Yes
29	Yes	Yes	Yes	Yes	Yes	Yes	Yes	Yes	Yes

**Figure 2:**
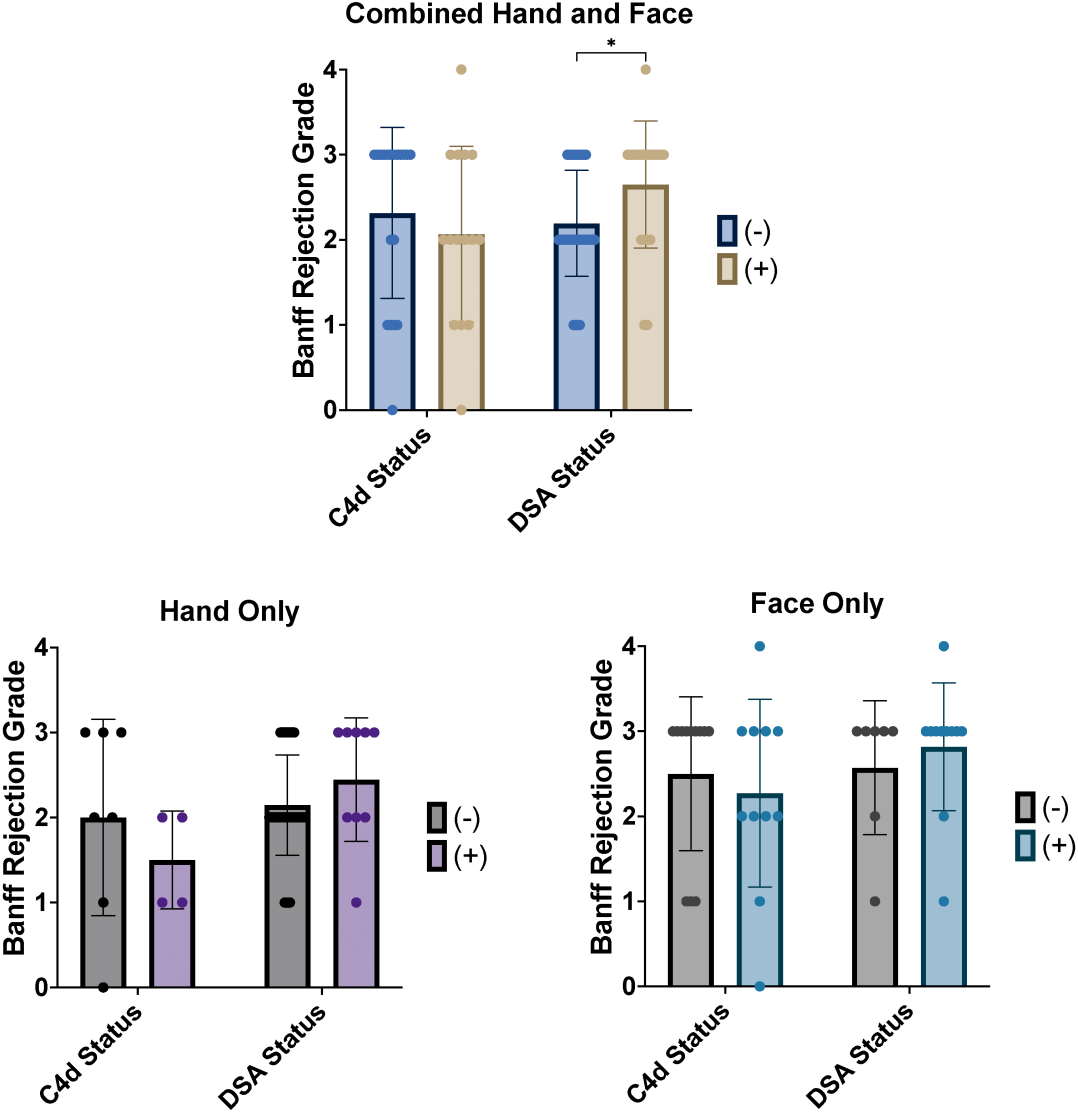
Acute rejection episodes of hand and face transplants classified by banff 2007 rejection grade with C4d and DSA status. For hand and face transplants combined, mean rejection grade was found to be significantly higher in episodes reported positive for DSA (*p* = 0.005) but was not found to be different between episodes reported positive or negative for C4d (*p* = 0.33). Mean rejection grade was also not found to be significantly different based on status of either marker for isolated hand or face transplants (Hand Only: C4d; *p* = 0.42, DSA; *p* = 0.15, Face Only: C4d; *p* = 0.50, DSA; *p* = 0.61).

When analyzing face and hand transplants together, rejection grade was found to be significantly higher in DSA positive episodes than in DSA negative episodes (*p *= 0.005, Figure 2), with mean rejection grades of 2.7 ± 0.7 and 2.2 ± 0.6, respectively. Positive or negative staining for C4d appeared to be non-specific with regards to the severity of acute rejection (*p* = 0.33, Figure 2). When analyzing face and hand transplants separately, no significant differences in mean rejection grade were found based on C4d or DSA status (Hand Only: C4d; *p *= 0.42, DSA; *p *= 0.15, Face Only: C4d; *p *= 0.50, DSA; *p *= 0.61, Figure 2). Finally, no significant association was found between the concurrent statuses of C4d and DSA (*p* = 0.23, Table 3).

**Table 3:** Acute rejection episodes.

	C4d (−)	C4d (+)
DSA (−)	10 episodes	5 episodes
DSA (+)	4 episodes	7 episodes

Acute rejection episodes where both C4d and DSA were assessed for hand and face transplants (*n* = 26). No significant association was found between the status of the two markers (*p* = 0.23).

## Discussion

This systematic review aimed to better understand the role of C4d and DSA in VCA acute rejection, and to provide insight into the meaning of these markers in the context of VCA. Isolated reports in the literature indicate that C4d and/or DSA evaluation may be useful in the detection of ABMR in VCA (19). However, continuous diagnostic and their reporting would be necessary in order to determine the exact roleof DSA and C4d in ABMR in VCA.

The herein presented review also outlines ABMR-related diagnosis and research in VCA. The currently available Banff classification does not provide guidance on the diagnosis of ABMR and the exact role of antibody mediated allograft changes is poorly understood. Grading of rejection in VCA is performed based on the Banff scale based on the magnitude of immune cell infiltration and its proximity and effect on the dermal/epidermal junction. Such changes of cellular infiltration are seen at the time of DSA/C4d positivity. ABMR in VCA is often defined as a rejection of the allograft with immune cell infiltreation (graded on Banff scale) with concomitant evidence of DSA/C4d (39).

There is evidence that chronic rejection related changes (in SOT often thought to be related to chronic antibody mediated endothelial damage, such as vasculopathy and consecutive fibrosis of graft) may be related to long-term cellular rejection (40). For example, in a case published by our group, chronic graft changes were seen in the absence of graft vasculopathy (41). This may have been also due to sampling error or rejection within the graft that did not manifest in skin biopsies. Hence, the exact role of donor specific antibodies and C4d in the setting of VCA rejection is unclear.

Mechanistically, DSAs can impact different cell types of VCA grafts, in particular endothelial cells. Via HLA surface binding, DSAs can induce a pro-inflammatory state in endothelial cells (sub-lytic MAC deposition, induction of pathways that lead to activation and increased expresion of leukocyte adhesion molecules) (42). These adhesion molecules include ICAM-1 which was previously shown to be upregualted at the time of suspected antibody mediated VCA rejection (42).

Based on the herein presented data, rejection severity as graded on the Banff scale for acute T-cell mediated rejection may be higher in hand and face transplant rejection episodes at the time of donor specific antibody presence. While this was not statistically significant with hand and face transplants analyzed separately, mean rejection grade was still numerically higher for DSA positive episodes in both patient cohorts, with a clear trend towards significance in hand transplant patients. Additionally, status of C4d staining was not found to be associated with higher or lower rejection grade in either the combined or separate patient cohorts. This review also did not find a significant association between C4d and DSA status.

A longitudinal analysis of the same patients immunological characterizations conducted by Win et al. revealed insights in gene expression patterns during ABMR, TCMR and in the absence of rejection (39). Overexpression of endothelial-associated genes such as ICAM-1/LFA-1 and VCAM-1 was seen during ABMR.

**Figure 3:**
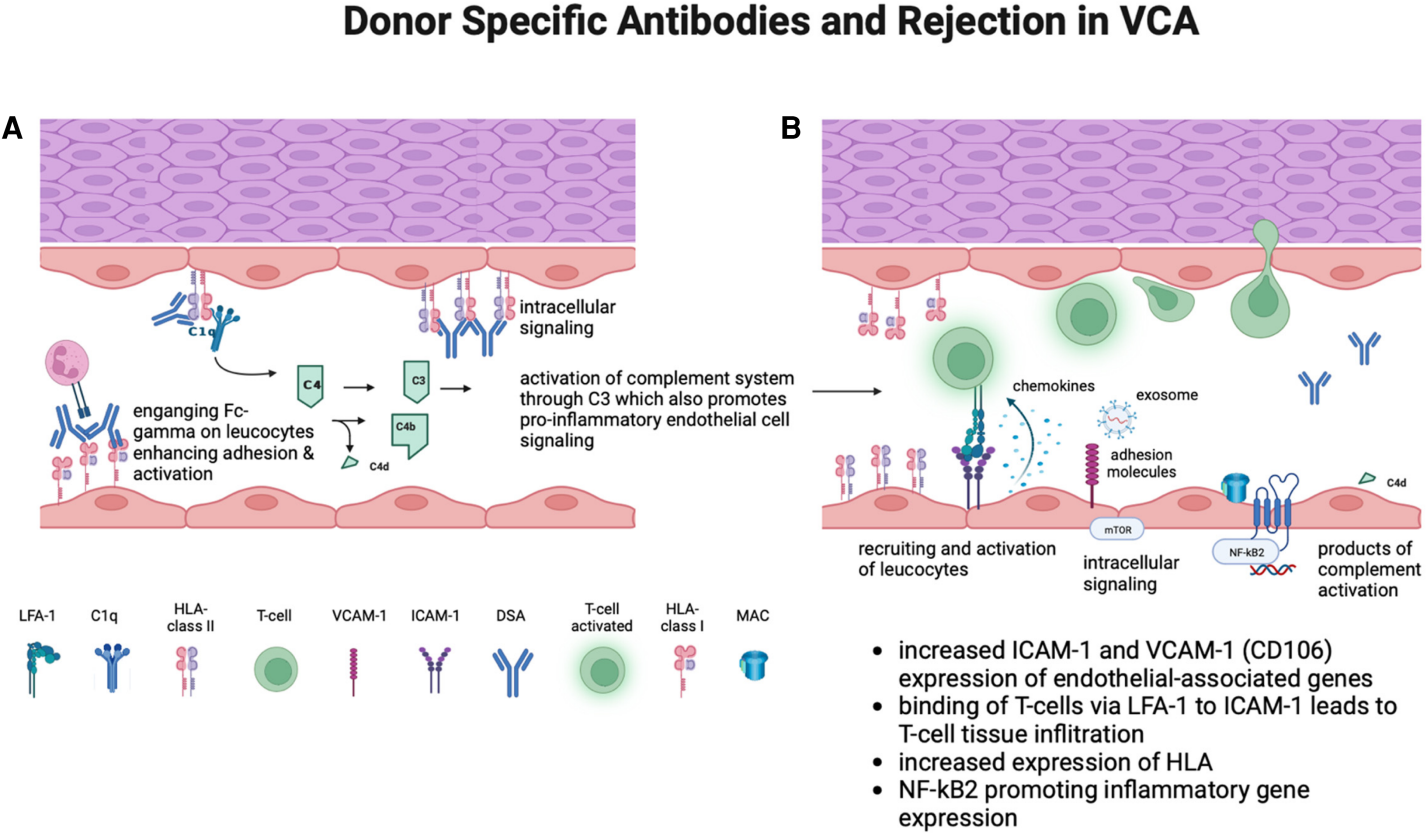
(**A**) ABMR is thought to primarily involve the graft endothelium and to be mediated by endothelial binding of anti-HLA antibodies (donor specific antibodies) against HLA surface molecules expressed on endothelium that lines donor vasculature. This can lead to a chronic inflammatory process ultimately leading to allograft vasculopathy (43). (**B**) In VCA, DSA may lower the threshold for homing of alloreactive T-cells, thereby promoting acute cellular rejection. This may be mediated by increased expression of leukocyte-endothelial cell interactions via ICAM1, VCAM1, and Sele (increased expression seen in suspected ABMR of VCA) (39). (Created with Biorender.com).

Based on these findings, it is possible that DSAs may lower the threshold for homing of alloreactive T cells, hereby facilitating acute cellular rejection (Figure 3). Additionally, based on our experience we have not observed a pure neutrophil and complement-mediated or antibody mediated rejection picture in skin, as may be seen in a rejecting kidney.

A multicenter study of hand transplantation (*n* = 44) found that the presence of alloantibodies, including DSAs, was associated with a greater cumulative incidence of rejection episodes grade II or higher (13). This is in line with the present finding that rejection grade is significantly higher in episodes where DSA are detected. Taken together with the findings of Berglund et al., there is growing evidence to suggest that the presence of DSA may be associated with worse acute rejection in composite tissue transplants.

Prior reports of C4d deposition in VCA have suggested that positive staining may be non-specific, or not associated with the presence of circulating DSA (16). Our finding that C4d alone does not predict the severity of acute rejection episodes is in line with these reports. However, while an association between C4d and DSA was not statistically significant, we believe that this relationship merits further investigation. The trend towards an association between concurrently positive C4d and DSA status suggests that there may be a relationship between these markers in VCA acute rejection. This review has revealed that reports of VCA rejection concurrent with assessment of both C4d and DSA are rare, and grade 0 rejections are almost never presented along with these markers. More detailed reports of rejection episodes are needed in order to assess the possibility of a relationship between these two markers, especially given prior findings in SOT.

The current body of literature describing VCA outcomes is quite limited given the novel nature of these surgeries and the small number performed thus far. The format of reporting antibody mediated acute rejection episodes is highly variable between VCA centers, which made the assessment of published data challenging. Furthermore, absence of a standardized guidelines complicates the comparison of regular follow-ups and outcomes between VCA centers. In order to address this, we only analyzed rejection episodes graded by the Banff classification 2007. Additionally, C4d and DSA were neither routinely assessed nor reported by most centers. As the field of VCA continues to develop and reports of rejection become increasingly available, greater investigation of the role of and relationship between of C4d and DSA should remain a question of interest.

As mentioned earlier, standardized guidelines in this regard are often lacking, which underscores the need for their development and integration into clinical practice. Incorporating C4d and DSA monitoring into routine follow-up protocols holds the potential to greatly enhance the early detection of rejection episodes and guide appropriate intervention. These minimally invasive tests offer a non-intrusive means of assessing the immunological status of VCA recipients, allowing for timely adjustments in immunosuppressive therapy. By establishing these markers as standard practice within comprehensive guidelines, healthcare providers can optimize patient outcomes, minimize the risk of graft rejection, and improve the long-term success of VCA procedures.

However, the small sample size presented herein constitutes a limitation in assessing a potential correlation between C4d, DSA, and rejection grade in VCA. Specifically, low event rate increases the likelihood of type II error, which could result in a failure to detect a significant relationship. Furthermore, we acknowledge differences between face and hand transplantation, particularly in the context of mucosal involvement in face transplants. However, due to the limited number of patients, we chose to aggregate these transplant out.

Additionally, our findings cannot serve as conclusive evidence of the absence of an association, as this may simply be due to a lack of statistical power. A lack of universal consensus on standardized guidelines in the follow up of VCA patients could be a primary factor contributing to the absence of significant findings in most comparisons. Finally, it is important to note that the presence of allorejection may be possible despite negative skin biopsies and therefore further investigations into non-invasive biomarkers for rejection in VCA may be emphasized, given the potential limitations of using skin biopsy for surveillance.

In conclusion, the presence of DSA appears to be associated with significantly higher rejection grades on the Banff scale of acute rejection for VCA. Association between C4d and DSA may lack significance due to various factors, such as a scarcity of data and low case numbers in the specialized field of VCA. To address these challenges, it is imperative to establish standardized guidelines for evaluation and diagnosis of acute and chronic rejection, emphasizing the pressing need for further research in this area. There is a lack of integration of gene expression analysis as it is standard for SOT (e.g., kidney). It is worth noting that the ease of conducting minimally invasive tests for C4d and DSA underscores their potential as valuable diagnostic tools for VCA patients, provided that robust protocols and guidelines are in place. These efforts are essential to improve outcomes and enhance the quality of care for individuals undergoing VCA procedures. Future work could also investigate the effect of different immunosuppressive regimens/rejection treatments on patient outcomes, as this may also interplay with C4d and DSA in episodes of acute rejection.
